# Social mobilisation, drug coverage and compliance and adverse reactions in a Mass Drug Administration (MDA) Programme for the Elimination of Lymphatic Filariasis in Sri Lanka

**DOI:** 10.1186/1475-2883-6-11

**Published:** 2007-11-15

**Authors:** Mirani V Weerasooriya, Channa T Yahathugoda, Darshana Wickramasinghe, Kithsiri N Gunawardena, Rohan A Dharmadasa, Kanchana K Vidanapathirana, Saman H Weerasekara, Wilfred A Samarawickrema

**Affiliations:** 1 Filariasis Research Training and Service Unit, Faculty of Medicine, University of Ruhuna, Sri Lanka

## Abstract

**Background:**

In Sri Lanka filariasis is endemic in Southern, Western and North Western provinces covering eight districts designated as implementation units in the Programme for the Elimination of Lymphatic Filariasis (PELF). Despite control activities over sixty years including multidose diethylcarbamazine, 6 mg/kg treatment microfilaria rates had persisted at low levels. Following systematic social mobilisation the first MDA with DEC albendazole combination was conducted in 2002.

**Methods:**

We investigated the extent social mobilisation had reached the people, their drug compliance and adverse reactions. Three localities were selected from each district to pick target population samples for pre-tested questionnaire. Three teams each with six people visited one district each day. One team worked from three starting points in one locality. A member applied eight part questionnaire to one family member totalling 150–160 people from one locality. Questions included social mobilisation, drug compliance and adverse reactions.

**Results:**

Information was disseminated by television, radio, banners and leaflets, to a lesser extent by people. Information reached more people in the periphery than in Colombo. 35.2% from Colombo municipality were unaware of the MDA. Drug coverage was 79.6%, home delivery 71.7% and delivery centres 7.9%. 35.6% in Colombo district and 53.4% from Colombo municipality did not receive drugs. Drugs were consumed by 71.4%. 28.6% who did not comply included 20.4% who did not receive them. 91.4% showed no adverse reactions, 7.5% were mild, 1.1% recovered with home remedies.

**Conclusion:**

Drug compliance showed significant positive correlation with awareness of the MDA. Door to door delivery was more successful than delivery from centres. More delivery centres conveniently located would have rectified this disparity. Poor awareness and compliance in Colombo and urban areas could be rectified with separate strategy for urban areas. More time for MDA and trained adequate manpower would ensure coverage to achieve elimination.

## Background

**Figure 1:**
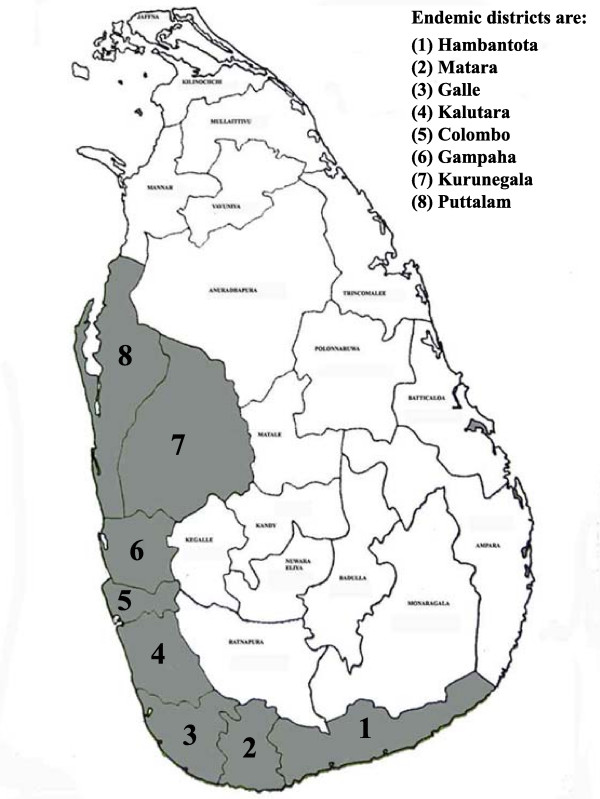
District map of Sri Lanka with the endemic area (districts) shaded.

Lymphatic filariasis is one of the major vector borne diseases in Sri Lanka, a disabling and disfiguring infection causing social stigma and economic reductions in life opportunities. The infection prevalent in Sri Lanka is *Wuchereria bancrofti*. The national control programme has recognised three provinces, Southern, Western and North Western as the areas where the disease is endemic on the island [1] (Fig. 1). The area is about 500 sq miles in extent and has a population of 9.8 million at risk. The land is relatively flat sloping towards the sea and is traversed by several streams and rivers. Much of the area is in the wet zone receiving a rainfall from the southwest monsoon.

In Sri Lanka despite control activities over a period of fifty years microfilaremia rates persisted at a low level. Selective treatment with multi-dose regimens of diethylcarbamazine (DEC), 6 mg/kg daily for consecutive days [2] on microfilaria (mf) carriers was the policy in operation at the time. The introduction in the late 1990's of mass treatment with single dose DEC and subsequently in combination with albendazole has become a landmark in the treatment and control of lymphatic filariasis. The single dose combination of DEC (6 mg/kg) and albendazole (400 mg) is now accepted as the treatment of choice for mass drug administration (MDA) in countries outside Africa where onchocerciasis is co-endemic with lymphatic filariasis [3]. Recent advances in methods both for controlling transmission and for simple and successful approaches for morbidity control [4] along with improvement in techniques for diagnosing infection [5] resulted in the World Health Assembly in 1997 to call on the WHO to initiate programmes to eliminate lymphatic filariasis as a public health problem in endemic countries [6]. Supporting this action SmithKline Beecham (now GlaxoSmithKline), commencing 1998, committed to donate via WHO albendazole to ensure the success of the elimination programme until 2020 [6]. Given this background the health ministries of countries with lymphatic filariasis established national elimination programmes [7].

### Programme for the Elimination of Lymphatic Filariasis

Sri Lanka set up a national Programme for the Elimination of Lymphatic Filariasis (PELF) in 1999 [8] based on the protocols recommended by the WHO [9]. The Ministry of Health identified the eight districts in three provinces where the disease is endemic as the smallest implementation units (IUs), which would be responsible for the administration of the PELF. An information, education and communication (IEC) campaign was set up before the MDA programme. The Ministry with the assistance of the World Health Organisation in 2002 prepared an intense and systematic social mobilisation programme called Communication-for-Behavioural Impact (COMBI) to support MDA efforts in Sri Lanka [10]. This programme used: (a) print media which utilised newspapers for articles and announcements, leaflets distributed to people and posters, banners and sign boards displayed in public places (b) electronic media where periodic television and radio programmes and short messages were transmitted. The topics included the disease and its symptoms, transmission of the parasite by the mosquito, treatment including single dose mass treatment with the combination of DEC and albendazole to be given annually for five years on the national 'filariasis day', the day assigned by the government for drug delivery and the additional benefits of albendazole, adverse reactions and prevention, morbidity management and control.

A vigorous and extensive training programme was carried out by the trainers to educate staff engaged in the Programme on all aspects of the disease, treatment with single dose MDA, how to conduct drug distribution and how to advise the target population on adverse effects and its contraindications. Each category and level of staff received training.

The PELF is under the responsibility of the Director of the National Filariasis Campaign who is the Programme Manager under whom medical officers and public health personnel carry out the programme. Under a decentralised system the programme is conducted in each IU in the three provinces through the Provincial Directors of Health (PDHs), and other public health staff under each Provincial Director of Health. The health personnel involved are mainly the public health nurses, public health inspectors and family health workers (FHW) formerly known as midwives who attend to the health and midwifery matters of the community.

The Programme Manager and the heads of the IUs obtained the required number of doses of the drugs, DEC, in the form of 50 or 100 mg tablets and albendazole in 400 mg tablets based on the census population, from the Ministry's central drug stores and allocated them to delivery staff through the MOHs a few days prior to the date of the MDA. The dosage of DEC was 300 mg for adults and 150 mg for children approximately to 6 mg per kg body weight. Drug delivery was conducted by two methods, door to door by deliverers visiting households and through delivery points and health centres from where people collected their drugs. In order to assist in drug delivery the MOHs in the IUs recruited volunteers among young people in the villages. Door to door delivery was conducted by volunteers, FHW and other health personnel. Each deliverer had to cover about 50 households.

The Programme is being monitored at different levels of implementation. These include availability of supplies at every level, availability of staff in adequate numbers and their training, completed efficiency reports to the higher administration levels, regular assessment of drug requirements and active surveillance for adverse reactions.

The first MDA in the national PELF covering the entire endemic region in 1999 using DEC, reported by the programme management [1], delivered the drug to only 62.7% of the population. In 2000 two rounds of MDA, again using only DEC, reported coverage of 68.2% in April and 70.5% in November. In the May 2001, when a limited supply of albendazole was available, the combination of DEC and albendazole was administered as a trial only in the Colombo district and achieved coverage of 76.7%. DEC alone was administered in the other seven districts. Following the implementation of the COMBI programme the first countrywide MDA using the two-drug combination was carried out in July 2002 with a reported coverage of 80% of the population. An independent evaluation of this programme was conducted in two localities in the Galle district [11].

The second round of the MDA with the two-drug combination was carried out on 27 July 2003 with a continuing social mobilisation campaign. An independent evaluation of this MDA programme by the Filariasis Research Training and Service Unit, Faculty of Medicine, University of Ruhuna, Galle to cover all IUs of the endemic region was carried out during 12 – 23 August 2003 after the final "mopping up" drug delivery. The observations of the IEC and drug compliance are presented in this report.

## Methods

### Study areas

**Figure 2:**
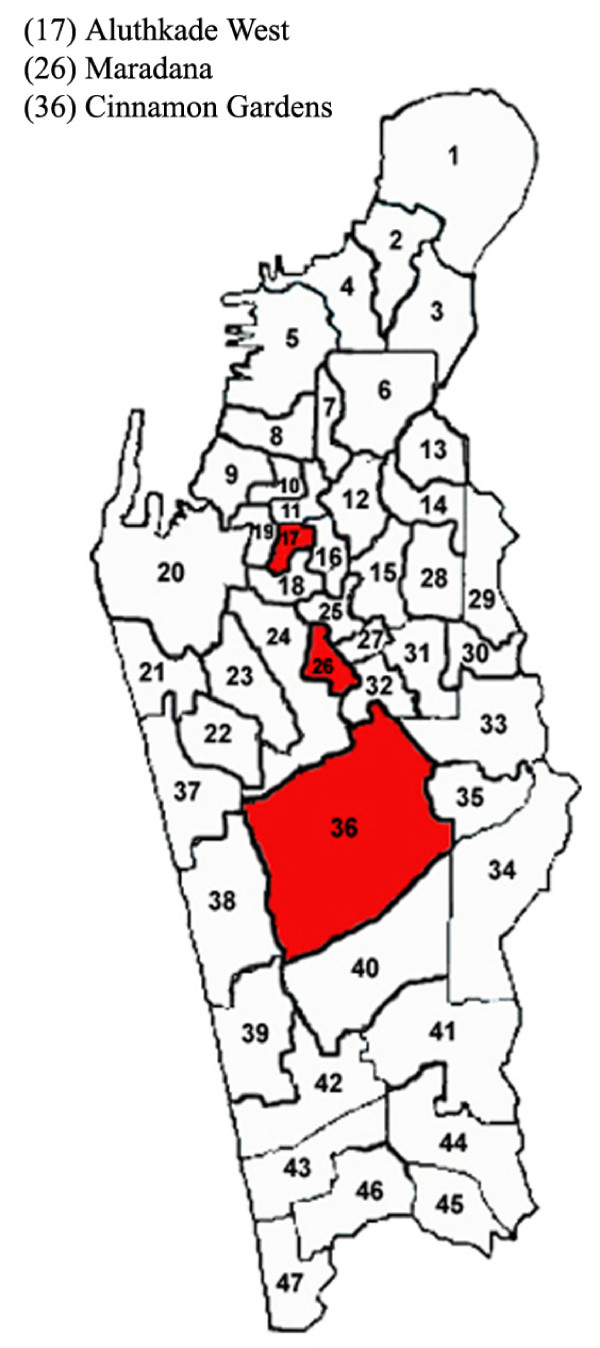
Ward map of Colombo Municipality area showing the localities investigated.

The eight districts included in the MDA programme were Hambantota, Matara and Galle in the Southern Province, Kalutara, Colombo and Gampaha in the Western Province, Kurunegala and Puttalam in the North Western Province (Fig. 1). The population of the area covering these districts is 9.8 million according to the Census of Population and Housing-2001. Three localities were selected from each district – mainly urban and suburban areas placed some distance from one another. In addition localities in three wards in the Colombo Municipality on the basis of housing and living standards of the population were selected. These localities are shown in Fig. 2.

### Method of house selection

Three teams participated in the survey. Each team consisted of six persons; five trained assistants and a doctor. The three teams worked in one IU on a single day, each team covering a single locality. In a locality the team selected three starting points along roads and streets placed some distance away from one another and two assistants commenced work from one starting point. They visited households on either side of the selected road. The houses which were closed were not surveyed. The questionnaire was applied directly to one adult from a family. Where two families lived in the same household one from each family was interviewed. Each assistant interviewed about 30 people, covering about 50 to 60 households along each road with an aim of a team covering about 150 to 160 people in the locality.

### Questionnaire

The pre-tested questionnaire had eight questions. The first was to ascertain how far the social mobilisation programme had reached the people and what the most successful method was in each locality. The second was on drug delivery, whether the drugs were delivered to the homes and by whom or whether they were collected at delivery centres. The third was to ascertain whether the drugs were delivered to them, on the national "Filariasis Day", in this instance Sunday 27 July, or later. The question of drug compliance was most important. The fourth and the fifth questions were whether the drugs were swallowed and the time the people swallowed them. In the sixth question we sought the reasons for not consuming the drugs from those who failed to do so. The seventh concerned the adverse effects following drug consumption, their degree of discomfort and whether they affected their day-to-day activities. The final question was whether the people knew about the purpose of the MDA. Ethical clearance for this study was obtained from the Ethics Committee, Faculty of Medicine, University of Ruhuna, Galle, Sri Lanka.

### Analysis

The data from the responses to questions in the questionnaire in each district (IU) were validated and analysed using SPSS 7.2 computer software (SPSS Inc., Chicago, IL, USA). Any association in the response to MDA between localities and districts was examined using the Chi square test.

## Results

A total of 4,358 people from 2,830 households were interviewed from 27 localities in the 8 districts and from three wards in the Colombo Municipality averaging about 160 people in about100 households from each locality. Details of population samples, their percentage of the District population and the number of households visited are given in Table 1.

**Table 1:** The population samples interviewed in eight districts (IUs) with a comparison of the observed and reported drug coverage in the independent evaluation of the July 2003 national MDA.

			Population sample interviewed				
					
Province	District (IU)	District Population (Millions)†	No of people	% District Population	% Observed drug coverage	% Reported drug coverage*	*P *value
Southern	Hambantota	0.53	490	0.09	96.3	93.0	0.30
	Matara	0.76	478	0.06	81.8	87.0	0.31
	Galle	0.99	470	0.05	80.2	83.0	0.61
Western	Kalutara	1.06	497	0.05	91.8	89.0	0.50
	Colombo	1.86	475	0.03	64.4	88.7	*P *< 0.001
	Gampaha	2.07	510	0.02	70.6	84.5	0.02
North Western	Kurunegala	1.45	477	0.03	89.5	82.0	0.13
	Puttalam	0.71	478	0.07	94.8	85.0	0.02
Colombo Municipality		0.38	483	0.13	46.6	88.7	*P *< 0.001
Total		9.80	4358	0.04	79.6	86.8	0.17

† Census of Population and Housing-2001, Department of Census and Statistics, Government of Sri Lanka, 2003.

* Source: Courtesy Programme Manager, National PELF.

The responses to each of the eight questions from all the localities were added together under each district. The people interviewed were of the age range 20 to 75 years. Females interviewed exceeded males in most localities in the ratio of around 3:2 mainly because interviews were conducted during working hours when men were away from home.

### Social mobilisation

**Figure 3:**
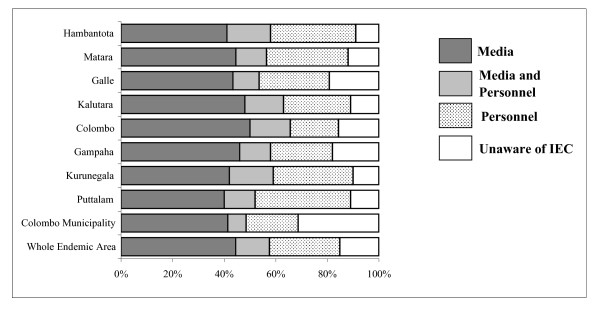
The extent of Information, Education and Communication (IEC) coverage in individual districts by different methods.

The social mobilisation programme by means of the IEC campaign about the MDA had been transmitted to the population by four media sources, television, radio, banners and leaflets (Fig. 3). The media message to the people interviewed had reached from 40.1% in Hambantota and Puttalam districts to 52.0% in the Colombo district. While the television and radio conveyed the message to most people in all districts, banners and leaflets were the most widely viewed message in Colombo district. Loudspeakers had also been popular in Kurunegala and Matara districts.

The FHW who form a regular cadre in the national Department of Health and volunteers had participated widely in the programme. Their participation ranged from 18.2% in the Colombo district to 32.2% in Kurunegala and Hambantota and 38.4% in Puttalam.

In the wards of the Colombo Municipality which were surveyed, 32.5% were not aware of the MDA programme. 19.2% in Galle district and 18.0% in Gampaha district were also unaware of the MDA programme. This is in contrast to the 15.6% in Colombo district (P < 0.001); 12.1% in Puttalam and Kalutara districts (P < 0.001); 10.1% in Kurunegala district (P < 0.001) and 9.2% in Hambantota district (P < 0.001). In the total sample from the endemic region 43.1% had the information through the media, 27.6% from the health workers and volunteers and 13.1% from the media and personnel. Whilst overall 16.2% of the people had not heard about the MDA.

### Drug coverage

We compared observed coverage with the reported coverage from the national MDA programme of June 2003 (see Table 1). Our observed coverage for the total area of 79.6% compared favourably with the reported coverage of 86.8%. The observed coverage of all districts except Colombo district and Colombo city wards sampled were not statistically significant and therefore consistent with the reported coverage. The observed coverage of 64.4% in the Colombo district and 46.6% in the Colombo city wards were low compared to reported coverage of 88.7% (*P *< 0.001) and 88.7% (*P *< 0.001) respectively.

**Figure 4:**
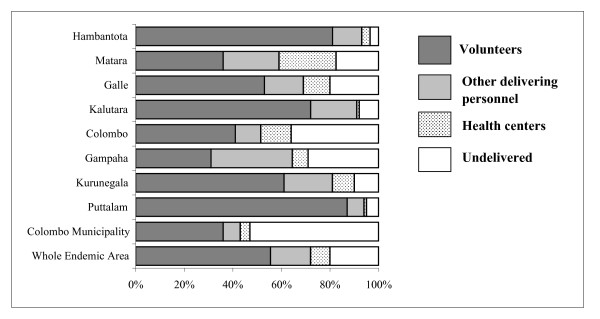
Observed drug coverage by different methods in individual districts.

Delivery achieved the highest coverage in Hambantota, Puttalam, Kalutara and Kurunegala districts (Fig 4). Door to door coverage was the more successful method of getting the drugs to the people largely undertaken by volunteers. The most effective delivery was by the volunteers in the districts of Puttalam (86.8%), Hambantota (80.6%) and Kalutara (72.2%). FHWs undertook drug delivery efficiently in the six districts they participated in, the highest being 17.4% in Matara. Delivery from centres had been poor. The only noteworthy figure was from Matara district where 28.9% of the interviewees collected their drugs from health centres.

Overall drug coverage had been poor in the districts of Colombo and Gampaha with 35.6% of the population in Colombo and 29.4% in Gampaha not receiving drugs. In the three wards in the Colombo municipality a worrying 53.4% of the people interviewed had not received the drugs. This was in contrast with the 3.7% who did not receive drugs in Hambantota (*P *< 0.0001) and 5.2% in Puttalam (*P *< 0.0001) and 8.2% in Kalutara (*P *< 0.0001).

In the sample interviewed 891 (20.4%) had not received drugs. Among 3,467 who received drugs 2,404 (55.2%) had them delivered to their homes by volunteers, 438 (10.1%) by FHWs and a further 281 (6.4%) by other health care personnel. Only 344 (7.9%) people received drugs from health centres. In all the 8 districts and in the Colombo Municipality between 71.1% and 95.3% of the people received the drugs on the "Filariasis Day".

### Drug consumption

Drug consumption had been highly successful in all districts. Most people who received the drugs had swallowed them. This group ranged from 83.3% in Colombo district and 94.6% in Kurunegala district. The wards taken up in the Colombo Municipality had a consumption rate of 89.1%.

In Galle, Matara, Kalutara, Gampaha and Colombo Districts over 95.0% of the people who swallowed the drugs had done so at night after meals. This proportion was over 82.0% in the other districts.

In all the districts 3,111 (71.4%) of the interviewees had consumed the drugs and 356 (8.2%) had not consumed them. 131 (3.1%) had refused drugs because they were under some other medication, 139 (3.2%) felt they did not need them, 48 (1.1%) had forgotten to consume them and only 38 (0.8%) were worried about adverse effects.

### Drug consumption vs. awareness of the MDA

**Figure 5:**
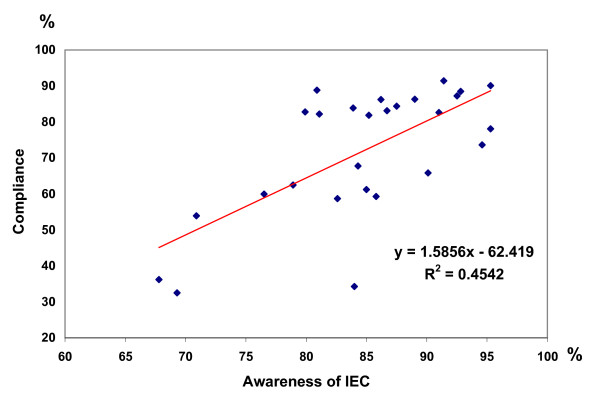
Relationship between awareness of IEC and Compliance.

There was a significant positive correlation between drug consumption and awareness of the MDA in all 27 localities visited (*P *< 0.0001) as shown by Fig. 5. Among the 3,111 who consumed the drugs 2,504 were aware of the MDA and 607 were ignorant of it. 219 of the 356 who did not comply were aware of the reasons for the MDA and 137 were not aware. Considering those who were aware of the MDA 91.9% of them had a valid reason for compliance (*P *< 0.0001) and 8.1% did not comply.

### Adverse reactions

The majority of the people interviewed in all districts and in Colombo Municipality had not reported any adverse effects. Of the total of 3,111 who consumed the drugs, 2,844 (91.4%) suffered no adverse reactions. Of the 269 (8.6%) who reported adverse reactions 79 (2.5%) complained of headaches and 34 (1.1%) complained of weakness. Other adverse reactions reported were dizziness 25 (0.8%), drowsiness 21 (0.7%), faintness 18 (0.6%), nausea 17 (0.5%), vomiting 15 (0.5%), fatigue 16 (0.5%), abdominal pain 12 (0.4%), headache and diarrhoea 13 (0.4%), headache and nausea 9 (0.3%), diarrhoea 6 (0.2%), fever 2 (0.1%) and rash 2 (0.1%). Severity was divided into three categories, mild (no interference with daily activity), moderate (interference with daily activity and recovery after home medication) and severe (seeking hospital admission). Among the 269 (8.6%) people with adverse reactions 236 (7.5%) were mild and 33 (1.1 %) were moderate. There were no severe cases, which needed hospital admission.

### Purpose of the MDA

A mean of 33.04% of people interviewed knew that the purpose of the MDA was elimination of the disease. They ranged from 22.0% in Galle district to 38.8% in Kalutara district. Gampaha district (38.4%), Hambantota (35.5%) and Puttalam (32.4%) recorded a high awareness about elimination. A higher proportion of the interviewees, 32.1% – 48.7% (mean 40.48%) thought the reason was prevention, the highest being in Puttalam district with 48.7%. Colombo, Matara, Kurunegala and Hambantata districts recorded over 40%. Between 18.8% and 38.5% did not know the purpose of the MDA. This ignorance was shown in 38.5% of the three wards in the Colombo Municipality investigated.

## Discussion

The present evaluation of an MDA in the national PELF was the first of its kind conducted by an independent group covering all eight districts in the endemic region. Our aim was to ascertain to what extent the IEC programme had reached the target population, the degree of success achieved in drug delivery and compliance and the extent of adverse reactions. Another independent survey [12] dealt with a target population in two *Grama Niladari *divisions and two clinics in the Gampaha district.

The IEC programme reached more people in the peripheral districts than in Colombo district and in Colombo municipality. The ignorance of the MDA shown by the interviewees of the Galle municipality area was reflected in the low awareness shown in the Galle district. In general, low awareness was evident in the urban areas we visited in all districts.

The combination of television, radio and loudspeakers was successful in carrying the message to 50.9% of the people whereas the percentage who had received the message verbally was 32.0%. This is in contrast to the situation in Leogane, Haiti [13]. Of the 91.2% respondents who knew about the MDA in Leogane, 55.7% heard about it from other people and 33.0% from radio broadcasts. In the Zanzibar programme [14] a combined message from an advisory board from ministries, national institutions, non-governmental organizations (NGOs), religious organizations and political parties was disseminated to the population. In Orissa, India [15] the IEC programme was run by district medical officers through the network of municipal health institutions and public health centres with the financial and technical assistance of the WHO.

Drug delivery strategies for MDA vary by country. In some programmes e.g. Leogane, Haiti [13] the public had been requested to collect their drugs from delivery centres. These centres were located in public places, geographically well distributed, convenient and easily located. In other countries delivery had been entirely door to door. In Zanzibar [14] trained 'filarial prevention assistants' delivered the drugs. In Orissa, India [15] trained community volunteers and peripheral health workers delivered drugs in the rural and municipal areas. In Tamil Nadu, India [16] delivery was implemented through the staff from the Primary Health Centres in the rural areas and through categories of health staff in the urban areas. In another instance in Tamil Nadu [17], they compared drug delivery by communities, community directed treatment (comDT) and health services directed treatment.

The national programme in Sri Lanka used two methods of drug delivery, door to door and from delivery centres. In our sample 79.6% of the eligible population had received the drugs. Door to door delivery far exceeded that achieved by delivery from health centres. Door to door delivery undertaken by volunteers and health care personnel including FHW was the more successful method. While drug coverage was good in the peripheral districts, in Colombo district and in the Colombo city our observations recorded poor coverage compared to reported coverage (*P *< 0.001). Similarly some urban localities in other districts also showed poor coverage.

In contrast much of the success of the Zanzibar MDA programme [14] was attributed to the drug distributors who were designated filarial prevention assistants (FPAs). They had been selected on the basis of their experience in public health activities and their residence in the community where the work was to be done. All distributors had T-shirts with logos for their identification. The training factor was highlighted in Orissa, India [15] where they found that in villages with planned training, delivery and compliance was high. Orissa [15] also recorded significantly lower levels of coverage and compliance in urban areas. They attributed this to the difference in the nature and composition of the urban population in addition to their higher levels of education, occupation and work pattern. Urban areas clearly need a different strategy to achieve a higher coverage and compliance to rural areas.

In the sample we interviewed 71.4% of the population had consumed the drugs. In the 28.6% who had not complied 20.4% had not received the drugs and 8.2% had not consumed them. The common reasons for non-delivery of drugs as observed in other programmes [15,16] were that the drug distributors did not visit the households or the family was out on the date of delivery. Often coverage could not be completed due to shortage of personnel and the time limit for delivery.

The reasons for their failure to consume drugs, apart from the fear of adverse reactions were the use of other drugs, not feeling the necessity for them and forgetting. Similar reasons have been given in other programmes [13,15,16]. All these reasons including some [16] such as, too many tablets needed to be consumed at one time, poor awareness of the benefits of the drugs and lack of confidence in the distributors could be eliminated through IEC to achieve the necessary behaviour change [15].

In some programmes the magnitude of adverse reactions affected coverage and compliance [14-16,18]. In Leogane, Haiti [18] 24% (17,421) of those treated during the MDA reported one or more adverse reactions. Among these 15,916 (91%) had minor adverse reactions which did not interfere with daily activities. Moderate adverse reactions, severe enough to interfere with activity were reported by 2% (1,502) of persons treated. In many places in Orissa [15] as a result of adverse reactions subsequent mopping up activities were suspended. In Zanzibar [14] the communities were educated to recognise adverse reactions as evidence of the therapeutic action of the drugs. As a result the public was willing to accept the drugs. Our observations in Sri Lanka showed that adverse reactions did not cause a significant problem. In the sample we assessed 91.4% had no adverse effects at all. Among the 8.6% who experienced adverse effects 7.5% were mild not needing attention and 1.1% had recovered after home remedies. The low adverse effects were probably due to low mf densities among the mf carriers.

We consider that strategies suited to the local situation are required to improve the MDA coverage and awareness of the people. Disseminating the message through people as mentioned above in Haiti and Zanzibar [13,14] could improve results. For example family health clinics and outpatient clinics could be utilised where doctors and health care personnel could address the people. Teachers could be given instructions on the MDA programme to be imparted to the children. The children would carry the message home to the wider population. Politicians in some districts who had shown commendable interest in the programme should be requested to help in carrying message to their constituents. The idea of seeking the cooperation from religious leaders could be explored especially in Muslim areas as Muslims will not traditionally welcome strangers in their homes. Wherever possible, the services of Lions Clubs and other NGOs should be requested both for IEC work and drug delivery.

The MDA programme needs increased human reservoirs for drug delivery. A "Filariasis Week" would be preferable to a "Filariasis Day" as delivery continued beyond one day. A serious shortcoming was identified with drug distribution centres as in some localities people had to walk several kilometres to reach one. There should have been an increased number of such centres, preferably located in every street in a given locality to enable people to reach them easily, as in Leogane, Haiti [13] with regular announcements giving the location of the centres.

The performance of volunteers varied by district. This was probably due to the level of training and appearance of the volunteers in some districts. Members of the public had no confidence in accepting drugs from some distributors. They lacked motivation and did not have adequate incentive as opposed to the drug distributors in Zanzibar [14]. Choice of personnel therefore becomes important. Selection of volunteers from medical students, nursing trainees and even year 12 school children would eliminate some of these problems. Training also becomes an important factor. Intensive training is essential for all drug distributors on communication skills, on the disease and its prevention. Therefore, the need for repeated and thorough training of volunteers and other categories of staff at all levels of the programme [9] cannot be overstressed. Introduction of mobile clinics equipped with loudspeakers would provide further improvement in drug coverage and mopping up operations.

## Conclusion

The study revealed that the level of awareness of the MDA and drug coverage in Colombo and urban areas needed to be raised appreciably to bring them in line with other areas. This could be rectified by better planning and by identifying a separate strategy for the urban population. Drug coverage showed significant positive correlation with awareness of the MDA. Door to door delivery was more successful than delivery from health centres. More delivery centres more conveniently located would have rectified this disparity. More time for MDA and trained adequate human resources would ensure coverage to achieve elimination.

## Competing interests

The author(s) declare that they have no competing interests.

## Authors' contributions

MVW and WAS conceived the study and developed the design, WAS and MVW prepared the manuscript, TCY carried out the statistics and prepared the figures and Table in addition to leading a team in the field. DW, NKG, RD, KKV and SHW lead teams in the field.
